# Autonomic and Vascular Responses during Reactive Hyperemia in Healthy Individuals and Patients with Sickle Cell Anemia

**DOI:** 10.3390/medicina59061141

**Published:** 2023-06-13

**Authors:** Erislandis López-Galán, Adrián Alejandro Vitón-Castillo, Ramón Carrazana-Escalona, Maylet Planas-Rodriguez, Adolfo Arsenio Fernández-García, Ileana Cutiño-Clavel, Alexander Pascau-Simon, Philippe Connes, Miguel Enrique Sánchez-Hechavarría, Gustavo Alejandro Muñoz-Bustos

**Affiliations:** 1 Departamento de Ciencias Básicas Biomédicas, Facultad de Medicina, Universidad de Ciencias Médicas de Santiago de Cuba, Santiago de Cuba 90100, Cubamaylet.planas@infomed.sld.cu (M.P.-R.); ileana.clavel@infomed.sld.cu (I.C.-C.); 2 Facultad de Ciencias Médicas “Dr. Ernesto Che Guevara de la Serna”, Universidad de Ciencias Médicas de Pinar del Rio, Pinar del Rio 20100, Cuba; adrianviton964@gmail.com; 3 Departamento de Ciencias Clínicas Básicas, Facultad de Medicina, Universidad Católica de la Santísima Concepción, Concepción 4090541, Chile; 4 Facultad de Ciencias Naturales y Exactas, Universidad de Oriente, Santiago de Cuba 90100, Cuba; adolfof@uo.edu.cu; 5 Hospital General “Dr. Juan Bruno Zayas Alfonso”, Laboratorio Vascular no Invasivo, Santiago de Cuba 90400, Cuba; alexander.pascau@gmail.com; 6 LIBM Laboratory, Team “Vascular Biology and Red Blood Cell”, Claude Bernard University Lyon 1, 69622 Lyon, France; 7 Grupo Bio-Bio Complejidad, Departamento de Ciencias Clínicas y Preclínicas, Facultad de Medicina, Universidad Católica de la Santísima Concepción, Concepción 4090541, Chile; 8 Núcleo Científico de Ciencias de la Salud, Facultad de Ciencias de la Salud, Universidad Adventista de Chile, Chillán 3780000, Chile; 9 Facultad de Ciencias de la Salud, Sede Concepción, Universidad de las Américas, Concepcion 4030000, Chile

**Keywords:** sickle cell anemia, pulse rate variability, pulse wave amplitude, autonomic nervous system

## Abstract

*Background and Objectives:* To compare autonomic and vascular responses during reactive hyperemia (RH) between healthy individuals and patients with sickle cell anemia (SCA). *Materials and Methods:* Eighteen healthy subjects and 24 SCA patients were subjected to arterial occlusion for 3 min at the lower right limb level. The pulse rate variability (PRV) and pulse wave amplitude were measured through photoplethysmography using the Angiodin^®^ PD 3000 device, which was placed on the first finger of the lower right limb 2 min before (Basal) and 2 min after the occlusion. Pulse peak intervals were analyzed using time–frequency (wavelet transform) methods for high-frequency (HF: 0.15–0.4) and low-frequency (LF: 0.04–0.15) bands, and the LF/HF ratio was calculated. *Results:* The pulse wave amplitude was higher in healthy subjects compared to SCA patients, at both baseline and post-occlusion (*p* < 0.05). Time–frequency analysis showed that the LF/HF peak in response to the post-occlusion RH test was reached earlier in healthy subjects compared to SCA patients. *Conclusions:* Vasodilatory function, as measured by PPG, was lower in SCA patients compared to healthy subjects. Moreover, a cardiovascular autonomic imbalance was present in SCA patients with high sympathetic and low parasympathetic activity in the basal state and a poor response of the sympathetic nervous system to RH. Early cardiovascular sympathetic activation (10 s) and vasodilatory function in response to RH were impaired in SCA patients.

## 1. Introduction

Sickle cell anemia (SCA) is an autosomal recessive disorder characterized by a single mutation in the β-globin gene, which leads to the production of abnormal hemoglobin (Hb) called HbS. When deoxygenated, HbS polymerizes, and a mechanical distortion of red blood cells (RBCs) occurs, i.e., sickling [1]. Sickle RBCs are fragile and very rigid, which makes patients prone to chronic hemolytic anemia, and frequent and recurrent painful vaso-occlusive crises (VOC) [1,2]. A wide variety of acute complications may occur in SCA, reflecting the complex pathophysiology of this disease [3].

It has been established that blood rheological alterations [2], chronic vascular inflammation, abnormal adhesion processes [3,4], and vascular dysfunction [5] play an important role in the occurrence of sickle cell anemia complications. One of the characteristic complications of sickle cell anemia are the vaso-occlusive crises, where it has been suggested that interactions between sickle erythrocytes and the vascular endothelium may contribute to its pathogenesis. Sickle red blood cells can occlude microvessels directly, by adhering to the endothelium, or indirectly, by disrupting endothelial functions. Interactions between sickle red blood cells, and the vascular endothelium and abnormal regulation of vasomotor tone may synergistically contribute to the occurrence of a vaso-occlusive crisis [6].

More recently, autonomic nervous system (ANS) dysfunction has been demonstrated to be implicated in the pathophysiology of SCA [7]. A recent study suggested that autonomic activity might be reduced in participants with sickle cell anemia during vaso-occlusion [8]. Inamo et al. [9] observed a lower mean ANS activity in SCA patients at baseline compared to healthy controls, but the degree of depression of autonomic activity greatly varied among patients. It has been suggested that there may be a relationship between autonomic reactivity and the clinical severity of patients with sickle cell anemia [10,11]. However, although ANS activity could be involved in the pathophysiology of SCA, the mechanisms through which an autonomic imbalance may modulate the clinical severity of disease are still poorly understood [12,13,14].

Several tests are available for the evaluation of the autonomic function, such as plasma catecholamines measurement, baroreflex sensitivity tests, and muscle sympathetic nerve activity [15]. However, these tests are expensive, time-consuming, and require well-trained personnel [16]. On the other hand, cardiovascular autonomic reflex testing (CART) has been considered the gold standard for the diagnosis of cardiovascular autonomic neuropathy (CAP), although some authors found this test difficult to apply and it requires the cooperation of patients [17]. ANS activity is commonly assessed through the quantitative assessment of heart rate variability (HRV), which reflects the sympathetic and parasympathetic control of the sinoatrial node [18,19].

Many works have found that pulse rate variability (PRV), which is extracted from photoplethysmography (PPG) data, can serve as an alternative measurement of ANS activity [20,21,22]. PPG is a simple and low-cost optical measurement technique that can be employed to detect blood volume changes in the microvascular bed of a tissue. Furthermore, it is generally accepted that PPG can provide relevant information about the cardiovascular system, such as heart rate, arterial pressure, stiffness index, pulse transit time, pulse wave velocity, cardiac output, arterial compliance, and peripheral resistance [22].

Reactive hyperemia (RH) is the increase in blood flow, within a given vascular territory, in response to an occlusion time [23]. Several factors have been implicated in the origin of this response, such as metabolic and endothelial vasodilators, and sensory nerves. RH is a relatively simple, reliable, and non-invasive test which is commonly used to assess endothelial function [24,25,26], but this test can also affect ANS activity, thus expanding its clinical applications [23]. RH can be quantified as a change in perfusion pressure, as measured by plethysmography [27]. However, to our knowledge, no studies have investigated the dynamic changed in ANS during RH in SCA patients. The present research aims to compare the variations in pulse wave amplitude, and autonomic and vascular responses during reactive hyperemia (RH) between healthy subjects and SCA patients.

## 2. Methods

### 2.1. General Description

A non-observational trial was conducted with 18 healthy subjects (HbAA genotype) and 24 patients with SCA (HbSS genotype) to study the effects of RH on vascular and cardiac activity. The sickle cell anemic patients were recruited at the Special Hematology Clinic of the “Juan Bruno Zayas Alfonso” General Teaching Hospital in Santiago de Cuba, during the 2013–2014 period. Before enrollment, all patients and control subjects had clinical examination with blood pressure measurements to check for the absence of reactive hyperemia contraindication. The participants were in a resting state for the first 10 min, under controlled ambient temperature (24–27 °C) and luminance (dim light) conditions. PPG was then used to record the pulsatile volume flow at the first finger of the lower right limb, both at baseline and during a post-occlusion hyperemia test. This research was approved by the Committee of Medical Ethics of Santiago de Cuba University of Medical Sciences and conducted in the Laboratory of Basic Sciences of the same institution. All subjects gave written informed consent and were in a clinical steady-state phase of their disease, with no painful crisis and blood transfusion within the previous three months.

### 2.2. Exclusion Criteria

The exclusion criteria were as follows: patients receiving treatment with hydroxyurea, central or peripheral nervous system disorders (stroke or neuromuscular disorders), skin disorders, implanted electronic devices (pacemakers or implantable cardioverter defibrillators), upper or lower limb amputations, women who were pregnant or menstruating, and any type of heart arrhythmia.

### 2.3. Physiological Recording

**Figure 1:**
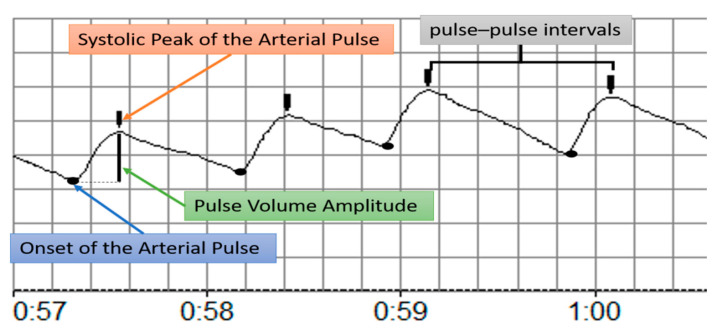
Schematic of physiological measurements in a photoplethysmography recording of the arterial pulse wave.

The PPG technique was used to record the arterial pulse wave in the first finger of the lower right limb using the ANGIDIN^®^ 18 digital plethysmograph (Biofísica Médica, Santiago de Cuba, Cuba), which captures the volume variations of the pulse wave via the reflection method (at 850 nm with a 3.5-mA photodiode). The PPG signal was then passed to an analog band pass filter from 0.1 to 15 Hz (Butterworth 5th order filter) and digitized at a resolution of 8 bits (106 samples per second). The recorded data were transmitted, processed and archived using the VAPLET^®^ auxiliary software (Biofísica Médica, Santiago de Cuba, Cuba). This software allows for the exportation of the signal in a beat-by-beat manner, such that it can then be processed by the Octave software 2019 (version 5.1.0; https://www.gnu.org/software/octave/download.html (accessed on 5 January 2021)) (Figure 1).

The onset and systolic peak of the arterial pulse wave were detected to calculate the pulse–pulse intervals (PPIs) and pulse volume amplitude (PVA) using the model proposed by Pascau-Simón [28]. The sphygmomanometer cuff remained fastened to the lower right limb to induce an arterial occlusion. The entire recording period comprised a 2 min baseline phase, 3 min arterial occlusion phase, and 2 min post-cuff deflation phase (RH phase). After recording the PPG signals during the 2 min baseline phase, an arterial occlusion was induced in the test limb using supra-systolic cuff pressure (50 mmHg above the subject’s baseline systolic pressure); signal measurement continued throughout the occlusion period. Occlusion was confirmed by constantly verifying the absence of a pulse waveform signal from the computer display. The cuff pressure was released completely after 3 min of arterial occlusion and the recordings continued during RH. There were no complications in the SCA patients and healthy subjects during the test performance. All measurements were obtained by the same person.

To establish the PRV, the PPIs of high-frequency (HF: 0.15–0.4) and low-frequency (LF: 0.004–0.15) bands were analyzed, as recommended by the International Consensus of Experts on HRV (1996) [29]. Time–frequency methods allow for changes to be tracked during the spectral analysis of HRV. Continuous wavelet transform (Morlet wavelet; CWT-Morlet) was used to calculate the PRV [30]. The results of the CWT-Morlet analysis were exported as a “.txt” file. A matrix of the results was generated for each subject (based on 2 min of recording) and interpolated at 2 Hz (0.5 s); in total, there were 240 PRV values.

### 2.4. Statistical Analysis

**Figure 2:**
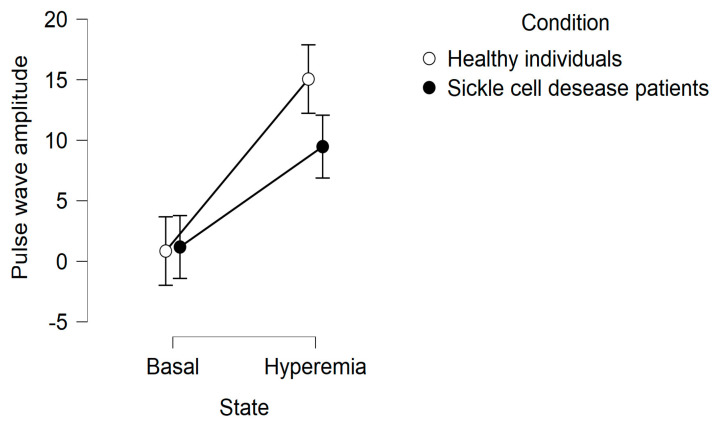
Pulse wave amplitude in the basal state and during the post-occlusion hyperemia test.

**Figure 3:**
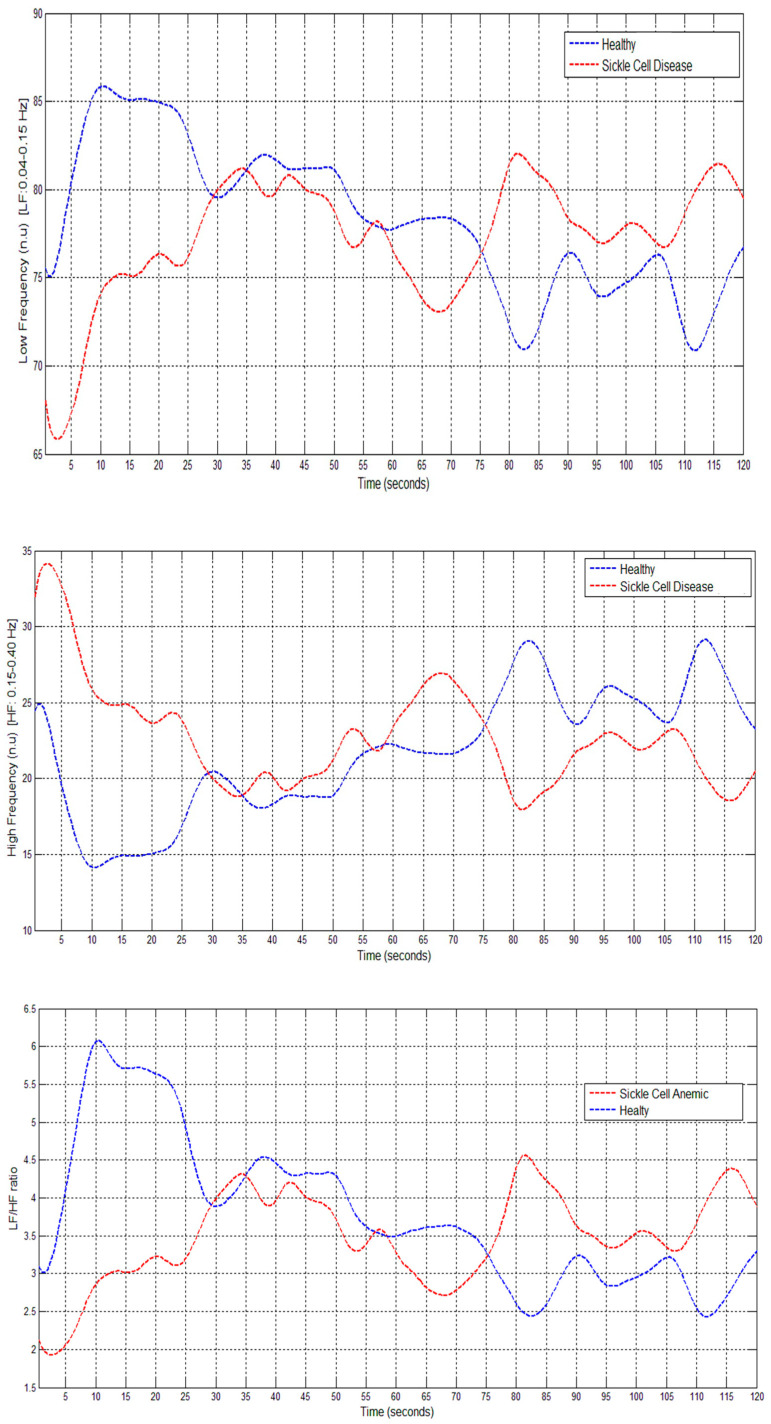
Dynamic autonomic response: time–frequency analysis of pulse rate variability during the post-occlusion reactive hyperemia test in healthy individuals and SCA patients.

For the comparison of the groups, a *t*-test of independent samples was applied for the variables that presented a normal distribution and the Mann–Whitney U test for those that did not. The data are expressed as the mean ± standard deviation (SD). Mathematical analyses and dynamic graphs (Figure 2 and Figure 3) were performed and generated, respectively, using the Octave software 2019 (version 5.1.0; https://www.gnu.org/software/octave/download.html (accessed on 15 January 2021)). The average of the LF/HF matrices for the PRV was calculated from the CWT-Morlet analysis during the 2 min period after cuff deflation and interpolated at 2 Hz (0.5 s). Statistical analyses were performed with the JASP software (version 0.16; https://jasp-stats.org (accessed on 31 January 2021)). Significance was defined as *p* < 0.05.

## 3. Results

The patients with SCA and the control healthy subjects were similar regarding age, sex, body mass index, systolic blood pressure (SBP), and heart rate at rest. The weight, height, diastolic blood pressure, and mean blood pressure levels were significantly lower in the SCA patients than in the controls (Table 1).

In the basal state, no statistically significant differences were found in the amplitude of the pulse wave between the healthy subjects and patients with sickle cell anemia. However, after reactive hyperemia, the post-occlusion pulse wave amplitudes were higher in healthy subjects compared to SCA patients (Figure 2). An increase in the LF/HF ratio was observed in healthy individuals at 10 s post-occlusion (early cardiovascular sympathetic activation). However, this was not observed in the SCA patients; instead, the LF/HF ratio increased 35 s post-occlusion and was higher than in healthy patients 75 s after cuff release (late cardiovascular sympathetic activation). The parasympathetic activity measured by HF was greater in SCA patients than in healthy controls after RH (Figure 3).

## 4. Discussion

In the present study, an impaired sympathetic activity in response to RH was observed in SCA patients. Previous studies have demonstrated that ANS activity is impaired in SCA patients compared to healthy individuals [7,31,32]. Nebor et al. [14] found lower parasympathetic activity and a greater sympatho-vagal imbalance in SCA patients with frequent pain crises than SCA patients without frequent pain crises and the control group. Charlot et al. [8] showed a higher sympatho-vagal imbalance in SCA patients during vaso-occlusive crises compared to steady-state, and that the length of hospitalization was related to the magnitude of the increase in ANS activity during the crisis.

Diastolic blood pressure (DBP) and mean blood pressure (MBP) levels were significantly lower in SCA patients than healthy control groups. These findings differ from what has been reported by other authors who have not observed such differences [7,32]. However, SCA patients had a higher SBP, DBP, and heart rate during vaso-occlusive crises (VOC) compared to steady state [8]. In addition, a greater vasoconstrictor response of neural origin has been demonstrated in SCA patients compared to healthy subjects, and that may play a role in the transition from steady-state to VOC [31]. In the present study, the patients were in a steady state, but with a sympatho-vagal imbalance, which may partially explain the lower levels of DBP and MBP.

The dynamic changes in ANS that occur during the activation maneuvers can be determined through the time–frequency methods used in the present study. The pattern of ANS activation during reactive hyperemia differs between SCA patients compared to healthy subjects. A quick sympathetic nervous system response, reflected in increments in the LF and the LF/HF ratio, was observed in healthy subjects after occlusion in this study. This has been interpreted as an early cardiovascular sympathetic reflex (10 s after occlusion) in response to RH caused by the metaboreflex [33]. The accumulation of metabolites during exercise has been shown to activate the metaboreflex [34,35]. In our SCA patients, although sympathetic activity in response to RH was impaired, it was greater immediately after the RH maneuver compared to the healthy controls, reflecting an imbalance in the ANS activity in these patients. Thus, time–frequency methods allow for the measurement of changes that might not be detected by classical methods.

In addition, the parasympathetic activity (i.e., HF) was greater in SCA patients than in healthy controls after RH. This indicates an exaggerated parasympathetic response that modifies reflexive sympathetic activity arising in response to RH in SCA patients. However, parasympathetic activity immediately after RH was lower in SCA patients than in healthy subjects. The low baseline levels of parasympathetic activity in these subjects may explain the inability of the heart to respond sufficiently to stimuli that activate the sympathetic nervous system.

Pearson et al. [10] speculated that autonomic dysregulation could alter the autonomic tone in SCA patients and may therefore exacerbate pain episodes by increasing peripheral vasoconstriction. Moreover, the sudden deaths of some SCA patients could be partly related to cardiovascular autonomic nervous dysfunction [11]. In patients with many forms of anemia, the decreased ANS activity and impaired sympatho-vagal balance has been attributed to chronic anemia. However, frequent-crisis SCA patients had lower parasympathetic activity than infrequent-crisis SCA patients, even though both groups exhibited a similar low hemoglobin concentration and hematocrit levels [14].

In rats, chronic intermittent hypoxia causes significant cell loss in the nucleus ambiguus, a structure from which several vagal efferent axons innervate ganglionated plexuses in the dorsal surface of cardiac atria, which in turn may have functional roles in cardiac regulation [36]. In addition, chronic intermittent hypoxia alters the structure of cardiac ganglia, resulting in the reorganization of vagal efferent projections to the cardiac ganglia [37]. Subclinical microvaso-occlusive and intermittent subclinical episodes of ischemia could contribute to the cardiovascular autonomic dysfunction observed in SCA [11]. The characteristic widespread vascular occlusions in SCA that cause chronic hypoxia can involve virtually every organ of the body, including the heart [38]. Moreover, cerebral thinning observed in patients with SCA may contribute to abnormalities of autonomic functions, and a hypersensitivity of the ANS responses to hypoxia may also be caused by the irregularities of brain function [39].

The results of the present study show that there was no significant difference between SCA patients and healthy subjects in pulse wave amplitudes in the basal state. It has previously been reported that increased peripheral blood flow occurs during the steady state with a periodic microcirculatory flow pattern, which may compensate for alterations in red blood cell rheology. The arteriolar diameter increases during the vaso-occlusive crisis compared to the intervals between the crises [7]. However, during the post-occlusion hyperemia test, SCA patients had small pulse wave amplitudes. This difference probably reflects the increased endothelial damage caused by repeated insults. The increase in pulse amplitude by hyperemia reflects a complex vascular response to ischemia [40,41]. Selvaraj et al. [42] reported that the PPG amplitude response reflected the metabolic component reinforcing the later course of RH. The results of the present study are consistent with the data of other groups examining endothelial dysfunction in SCA. Patients with sickle cell anemia had severely impaired endothelial function during crises [43] and in a steady-state phase [6] as compared to health-matched controls. Other studies reported that the group of patients who had more symptoms had a significantly lower RH index than the group that was not as symptomatic.

The hyperactive endothelium seen in SCA enhances red blood cell and neutrophil adhesion, resulting in a slowed blood flow and sickling in post-capillary venules, followed by vaso-occlusion and ischemia [44]. In addition, Suvimol et al. [39] demonstrated that autonomic dysregulation in subjects with SCA enhanced normal vasoconstriction reflexes. Global vasoconstriction increases microvascular transit time and may promote the entrapment of sickle cells in the microvasculature, making vaso-occlusion more likely [31].

Many articles have reported an alteration of ANS activity in response to RH in patients with SCA. However, these papers did not assess dynamic changes in the ANS during the RH maneuver. The dynamics of ANS activation during RH may provide valuable information for evaluating the cardiovascular autonomic neuropathic that may be present in sickle cell disease. The results of the present study demonstrate that both ANS activity and PPG amplitude are impaired in patients with SCA; thus, the autonomic and endothelial status in SCA patients can be closely evaluated by applying the techniques detailed herein. Further studies are clearly warranted to clarify the relationship between altered ANS activity and SCA severity. A limitation of our study was the small sample size. This was, however, comparable to many papers concerning ANS activity in the literature.

## 5. Conclusions

Vasodilatory function, as measured by PPG, was lower in SCA patients compared to healthy subjects. Moreover, a cardiovascular autonomic imbalance was present in SCA patients with high sympathetic and low parasympathetic activity in the basal state and a poor response of the sympathetic nervous system to RH. An early cardiovascular sympathetic activation (10 s) and vasodilatory function in response to RH were impaired in SCA patients.

## Figures and Tables

**Table 1:** Population characteristics of the healthy controls and SCA patients.

	AA (*n* = 18)	SS (*n* = 24)	*p*
Sex, male/female	7/11	10/14	0.856
Weight, kg	71.9 ± 9.27	53.25 ± 14.20	<0.001
BMI	26.47 ± 3.86	24.46 ± 7.76	0.077
Age	44.5 ± 11.6	43.29 ± 11.31	0.737
Height, cm	160 ± 0.07	150 ± 0.18	0.005
SBP, mmHg	121.66 ± 6.18	114.58 ± 15.03	0.068
DBP, mmHg	81.11 ± 6.76	73.75 ± 11.34	0.019
MBP, mmHg	94.62 ± 6.06	87.36 ± 11.67	0.021
HR, beats/min	78.17 ± 4.82	75.46 ± 8.46	0.311
Hb, g/L	-	76.71 ± 18.46	-
Ht	-	0.24 ± 0.059	-

AA, healthy controls; SS, SCA patients; *n*, number of subjects; SBP, systolic blood pressure; DBP, diastolic blood pressure; MAP, mean blood pressure; HR, heart rate; Hb, hemoglobin; Ht, hematocrit.

## Data Availability

Not applicable.
